# Multimodal Nature of the Single-cell Primate Brain Atlas: Morphology, Transcriptome, Electrophysiology, and Connectivity

**DOI:** 10.1007/s12264-023-01160-4

**Published:** 2024-01-09

**Authors:** Yuhui Shen, Mingting Shao, Zhao-Zhe Hao, Mengyao Huang, Nana Xu, Sheng Liu

**Affiliations:** 1 State Key Laboratory of Ophthalmology, Zhongshan Ophthalmic Center, Sun Yat-sen University, Guangdong Provincial Key Laboratory of Ophthalmology and Visual Science, Guangzhou, 510060 China; 2 Guangdong Province Key Laboratory of Brain Function and Disease, Guangzhou, 510080 China

**Keywords:** Neuron, Brain atlas, Patch-seq, Connectivity, Primate

## Abstract

Primates exhibit complex brain structures that augment cognitive function. The neocortex fulfills high-cognitive functions through billions of connected neurons. These neurons have distinct transcriptomic, morphological, and electrophysiological properties, and their connectivity principles vary. These features endow the primate brain atlas with a multimodal nature. The recent integration of next-generation sequencing with modified patch-clamp techniques is revolutionizing the way to census the primate neocortex, enabling a multimodal neuronal atlas to be established in great detail: (1) single-cell/single-nucleus RNA-seq technology establishes high-throughput transcriptomic references, covering all major transcriptomic cell types; (2) patch-seq links the morphological and electrophysiological features to the transcriptomic reference; (3) multicell patch-clamp delineates the principles of local connectivity. Here, we review the applications of these technologies in the primate neocortex and discuss the current advances and tentative gaps for a comprehensive understanding of the primate neocortex.

## Introduction

The mammalian neocortex is responsible for high-cognitive function and fine motor skills. The neocortex fulfills these functions through complicated networks of diverse neurons. Studies using rodents have established a basic framework of the neocortex, censused the transcriptomes of all the major cell types, and linked them to their physiological properties and principles of connection. Despite its augmented cognitive function, the primate neocortex shares the basic neuronal program with rodents. Accumulating evidence reveals the divergence between rodents and primates [1–3]. However, the distinctions are subtle. A multimodal census of the neurons of the primate neocortex is necessary to underpin evolutionary changes that augment the capacities of the primate neocortex.

Primates exhibit complex brain structures that augment cognitive function. However, the volume and number of neocortical neurons increased rapidly compared to subcortical structures during the evolutionary expansion of the neocortex [4–8]. While the general principles of cortical development and basic architecture are conserved, studies have shown differences in the cellular composition of the human cortex [9]. These differences include the expansion of superficial cortical layers during mammalian evolution, which may involve rare cell types and novel cellular interactions contributing to the complexity of primate brain function [10]. Notably, neurons such as von Economo [11] and rosehip neurons [12], which have unique morphological features, are primate-specific and do not exist in mice. These neurons may be involved in various cognitive processes, including facilitating rapid information transmission across different brain regions and promoting the integration of sensory, emotional, and mental information. In addition, there are transcriptional differences between mice, non-human primates, and humans, particularly in genes related to neuronal structure and function [2, 13–15]. For example, glutamatergic neuron transcriptome types are more diverse in the supragranular layer of the human neocortex [16]. The development of novel research technologies and massive high-throughput studies provide valuable resources for understanding the foundation of the augmented cognitive capacities of the primate brain.

First, it is crucial to systematically investigate the cell type composition within primate cortical areas. Since Frederick Sanger invented Sanger sequencing in 1977, when we could read the genetic code for the first time, it has taken several decades and significant efforts to promote the development of sequencing technology [17]. Due to its cost, sequencing became the conventional technology used in regular research until next-generation sequencing methods were announced in 2005, followed by the invention of the single-cell RNA-seq (scRNA-seq) method [18]. However, due to the limited application of scRNA-seq to frozen tissue with cell membranes ruptured during freezing, an essential complementary technology was developed that involved isolating a single nucleus sequencing the RNA (snRNA-seq) [19, 20]. This approach initiated a new chapter for investigating the cell type composition of primate cortical areas [21]. Previous neuronal classification was based on morphology, electrophysiology, or some specific molecules [22], while sc/snRNA-seq provides high-throughput analysis and single-cell resolution for the robust classification of cell types and generating a transcriptomic cellular atlas [23]. By comparing the cell type composition across species, primate-specific cell type composition and proportions might be evaluated to help explain the more complex brain functions of primates [24]. After generating a transcriptomic reference cellular atlas [25–37], thorough gene expression analysis might improve understanding of cell differentiation trajectories by identifying abundant related pathways and genes, which could serve as candidate targets for interventions targeting neurodevelopmental disorders [38–45]. When comparing physiological and pathological states, sc/snRNA-seq helps identify the modification of the composition and proportions of cell types with related pathogenic signaling pathways or genes, which can not only reveal the molecular mechanisms of pathological processes but also provide abundant promising targets for therapeutic protection and intervention [17, 46–49].

However, because of the multiple dimensions of the neuron, which is the basic unit of the nervous system, integrating the wealth of transcriptomic data with well-established morphological and electrophysiological data is still required [50]. Since Neher and Sakmann invented patch-clamp technology to study ionic currents at the single-cell level, this method has become the standard for investigating electrophysiology and morphology in single cells, especially neurons [51]. Genetically-labeled cells facilitate the study of the morphology and electrophysiology of neurons [52–55]. However, the application of these techniques is limited by the availability of known cell type-specific markers and the operational feasibility of primate experiments. Recently, several groups have developed and optimized Patch-seq, a multimodal method that describes electrophysiological, transcriptomic, and morphological profiles in single neurons of rodents [56–60] and adult human and non-human primate brain slices [16, 59, 61–65]. By combining this technology with innovative data analytical tools [66–68], neuroscientists can map the Patch-seq data to the transcriptomic reference atlas for assigning morphological and electrophysiologic annotations to enrich the transcriptomic cellular atlas to improve a comprehensive understanding of the primate cortical areas during physiological or pathological states [69–73].

After generating a transcriptomic cellular atlas with electrophysiological and morphological annotations, the next step is to analyze the connectivity principles within cortical areas. However, the currently popular technologies that identify neuronal types typically need more morphological data and heavily rely on genetic manipulation, which is challenging in primate experiments. To decipher the principles of local connectivity, a high-throughput technology and robust cell classification standards are critical [74–78]. Simultaneous multiple whole-cell patch-clamp recordings, such as dual, triple, and quadruple recordings, have proven invaluable in facilitating the study of connectivity between neurons [79, 80]. The number of test potential connections is significantly increased with simultaneous patch-clamp recording neurons. Multicell patch-clamp setups with up to 12 simultaneously recorded neurons were achieved before 2011 [81–83]. The stable simultaneous octuple patch-clamp recording technology achieved superior multicell patch-clamp recording results [84, 85]. Because it provided highly detailed morphological data from neurons for neuronal type identification and offered high-throughput evaluation of potential connections, it is optimally suited for primate research on connectivity and addresses concerns regarding the considerable workload in primate studies and the scarcity of primate tissues.

Here, we review the advances in primate neuroscience from the applications of the above three advanced technologies and discuss the potential of integrating the wealth of datasets obtained using those technologies to generate a primate brain atlas with multiple dimensions (describing transcriptomic, electrophysiological, and morphological profiles, as well as the principles of local connectivity) for comprehensively understanding the functional mechanisms of primate cortical areas. While sc/snRNA-seq can be used to generate a transcriptomic reference atlas of primate cortical areas with transcriptome-based classification, Patch-seq can provide morphological and electrophysiological annotations for the above transcriptomic reference atlas. In addition, the multicell patch-clamp measures the strength of monosynaptic connections between cells. These physiological properties can be mapped to the transcriptomic reference atlas, depicting a multimodal atlas of the primate brain, and facilitating the advancement of our knowledge in neuroscience.

## Large-scale Transcriptomic Analysis Advances Primate Neuroscience

**Fig. 1:**
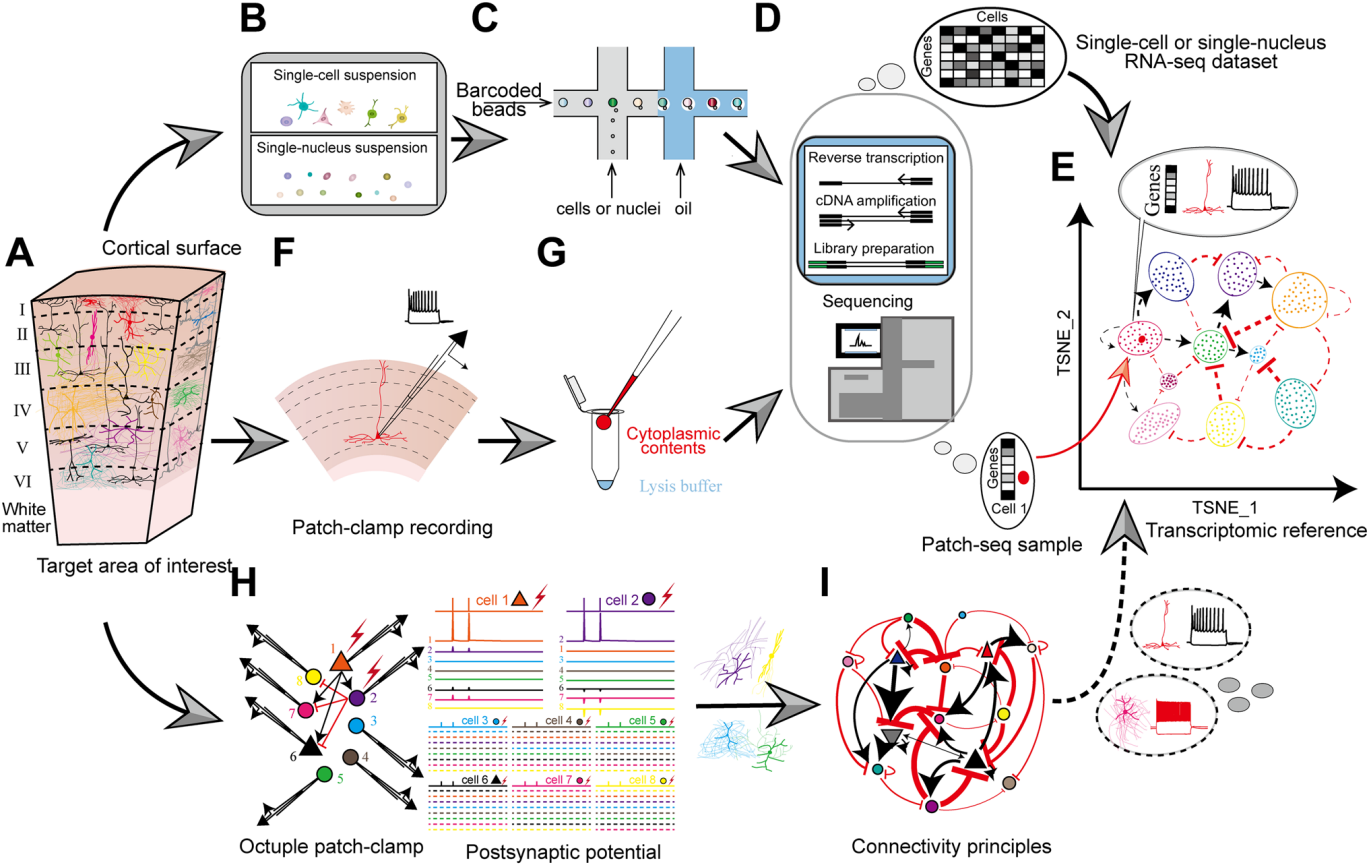
Schematic workflow of the three technologies for investigating the multimodal nature of the primate brain atlas. **A** Abridged general view of the organization pattern of the target area. **B–E** Schematic of a single-cell/single-nucleus RNA-seq experiment. **B** Preparation of single-cell/single-nucleus suspension. **C** Microfluidic device pairs individual cells or nuclei with barcoded beads that collect and barcode the cell's mRNAs in droplets. **D** The above droplets are then broken and reverse transcribed, amplified, and sequenced to generate a single-cell/single-nucleus RNA-seq dataset (upper). **E** This dataset establishes the transcriptomic reference atlas with transcriptome-based classification. **D–G** Schematic of Patch-seq experiment. **F** Patch-seq simultaneously performs electrophysiological recording on the acute slice and dye penetration for further staining and morphological reconstruction. **G** After electrophysiological recording, most of the cytoplasmic contents are aspirated using the patch pipette and transported into a lysis buffer. This pretreatment is followed by a standard single-cell RNA-seq protocol (**D**), including reverse transcription, amplification, and sequencing, and generates the Patch-seq sample (lower); the gene expression profile of the Patch-seq sample can be used to map the cell onto the above transcriptomic reference atlas (the red point in **E**), thus assigning electrophysiological and morphological annotations to the transcriptomically identified cell type. **E, H–I** Schematic of multicell patch-clamp experiments. **H** Connectivity diagram of the eight simultaneously-recorded neurons (triangles, excitatory neurons; circles, inhibitory neurons); In an octuple patch-clamp recording, 56 potential connections are tested (eight neurons are stimulated successively (left), and postsynaptic potentials of the other seven neurons are recorded for testing neuronal connections (right)). **I** Based on the morphological reconstruction and classification, connectivity principles within the selected cortical area can be delineated (black lines, excitatory connections; red lines, inhibitory connections; line thickness, connection probability). The dataset from multicell patch-clamp recording possesses the potential of mapping those connectivity principles into the above transcriptomic reference based on the shared morphological and electrophysiological message (dotted line with arrow in **E**).

Single-cell or single-nucleus RNA-seq technologies, which have high throughput and robust resolution for cell type classification with gene expression analysis during both physiological and pathological states, have provided an excellent opportunity for the in-depth exploration of the primate brain [18, 19, 86–88] (Fig. 1B−E).

### The Taxonomy of Transcriptomic Cell Types

Accumulating research on the rodent and primate cortex at single-cell resolution suggested that the cell types are largely conserved across species. The neocortical brain cells of primates are commonly analyzed to generate a taxonomy of cell types according to transcriptomic similarity. Brain cells are grouped into neuronal cells and non-neuronal cells. Neuronal cells are usually divided into glutamatergic excitatory neurons and GABAergic inhibitory neurons [89]. Each class can be further divided into multiple subclasses (Table 1). In the primate neocortex, such as the human middle temporal gyrus and macaque primary visual cortex, the excitatory neurons have been categorized into different subclasses based on the laminar distribution, transcriptome type analysis, and the transcriptomic homology to well-established datasets. These subclasses include intratelencephalic cell subclasses (L2/3 IT, L4 IT/IT, L5 IT, L6 IT, and L6 IT Car3), the L5 extratelencephalic cell subclass (L5 ET), the L6 corticothalamic cell subclass (L6 CT), the near-projecting cell subclass (L5/6 NP), and the L6b cell subclass [10, 89–93]. Alternatively, in a different system, based on the expression of marker genes, excitatory neurons are also divided into four subclasses: *CUX2*-expressing cells (or *LINC00507* [90], or *HPCAL*, combined with the application of the marker *NXPH4* to distinguish upper neurons from L6b neurons[91]) that are mainly located in the upper layers, *RORB*-expressing cells (enriched in layer 4 but can be found across all layers), *FEZF2*-expressing cells (located in deep layers), and *THEMIS*-expressing cells (located in deep layers). The GABAergic inhibitory class contains four main subclasses (*LAMP5*, *VIP, SST*, and *PVALB*-expressing) [94, 95]), essentially correspond to their developmental origin in the medial ganglionic eminence (MGE: *PVALB* and *SST* subclasses) or caudal ganglionic eminence (CGE; *LAMP5* and *VIP* subclasses) [15, 96, 97]. There are additional subclasses such as the *PAX6* [91, 98], *LAMP5 LHX6* [10, 91, 98], and *PAX6 ADARB2* (SNCG) [90, 98] subclasses originating from the CGE, and the *PVALB UNC5B* (Chandelier) [90, 98] and *SST CHODL* [90, 98] subclasses originating from the MGE. Non-neuronal brain cells are grouped into six subclasses: astrocytes, oligodendrocyte precursor cells, oligodendrocytes, microglia, and perivascular macrophages, endothelial cells, and vascular leptomeningeal cells [90, 91, 93, 94, 96, 97]. These neuronal and non-neuronal subclasses are subdivided into cell types based on additional marker genes.

**Table 1:** Primate Neuronal Subclasses and Morpho-electrical Annotations.

Class	Subclass (Transcriptomic reference)		Laminar distribution	Morpho-electric annotations by Patch-seq
GABAergic	CGE	*LAMP5*	Enriched in upper	
		*VIP*	Enriched in upper	
		*PAX6*	Enriched in upper	
		*LAMP5 LHX6*	Evenly	
		*PAX6 ADARB2 (SNCG)*	Enriched in upper	
	MGE	*PVALB*	Evenly	
		*SST*	Evenly	
		*PVALB UNC5B* (Chandelier)	Enriched in upper	
		*SST CHODL*	Evenly	
Glutamatergic	L2/3 IT	*(CUX2+, HPCAL+NXPH4-, LINC00507+)*	Layers 2/3	Human [16], macaque [91]
	L4 IT	*(RORB+)*	Enriched in Layer 4	
	L5 IT	*(FEZF2+)*	Layer 5	
	L5 ET	*(FEZF2+, SCN4B)*	Layer 5	Human [90], macaque [65, 90]
	L5/6 NP	*(FEZF2+, HTR2C+)*	Layer 5/6	
	L6 IT	*(THEMIS+)*	Layer 6	
	L6 IT *Car3*	*(THEMIS+, OPRK1+, KRT17+)*	Layer 6	
	L6 CT	*(FEZF2+, SYT6+)*	Layer 6	
	L6b	*(HPCAL+NXPH4+)*	Layer 6	

### Cross-Species Transcriptomic Conservation and Divergence

Recent comprehensive cross-species transcriptomic studies that have generated transcriptomic cellular atlases with abundant molecular signatures have revealed surprisingly well-conserved neuronal and non-neuronal types across different cortical areas among primates and rodents [10, 15, 99, 100]. Humans share with rodents an evolutionarily-conserved regulatory program involved in the process of neuronal development, which controls the specification, migration, and differentiation of GABAergic interneurons [101]. However, the primate-specific cell types [12] and the differences in homologous cell types, including proportions, laminar distributions, gene expression, and morphological features, are inestimable [102, 103]. For example, homologous thalamocortical neurons in the primate dorsal lateral geniculate nucleus, which convey visual information from the retina to the primary visual cortex (V1), are distinct from those in rodents [99]. Detailed single-cell transcriptome analysis sampling from non-human primate V1, novel cell types (the *NPY-*expressing excitatory neuron type and the primate-specific activity-dependent *OSTN+* neuron type), and different gene expression patterns have been revealed in the primary visual cortex in primates [91]. These findings may account for the high visual acuity and more complex color vision of primates.

Moreover, comparative RNA sequencing has revealed divergent expression patterns regulating cell morphogenesis, such as *ZEB2* (zinc finger E-box binding homeobox 2) and human-specific *NOTCH2NL* (paralogs of the *NOTCH2* receptor). The expression of *ZEB2* promotes the neuroepithelial transition and manipulations of the related downstream signaling lead to the acquisition of non-human ape architecture in the human context and *vice versa*. On the other hand, *NOTCH2NL* expands cortical progenitors and enhances neuronal output, emphasizing the important role of neuroepithelial cell shape in human brain expansion [104, 105]. These findings suggest that cell type taxonomy is largely conserved from rodents to primates, yet differences exist.

### The Transcriptomes During Brain Development and Neurogenesis

The primate brain, which has the largest volume relative to body size and ~1000 times more neurons than the rodent brain [106, 107], is much more complex. Single-cell and single-nucleus technologies have established cellular taxonomies of multiple cortical areas from developing primates using gene expression patterns. This considerably expands our knowledge of early neurogenesis, neuroplasticity, and cellular differentiation during the early developmental stage [108–117]. Transcriptomic data, which provides cell lineages, molecular signatures, and transcriptional regulatory networks that underlie the basis of physiological activities [118–122], can be used to assess human and non-human primate brain development and early neurogenesis [105, 107, 123, 124]. Moreover, synaptic gene expression patterns show considerable differences in human cortical areas during aging, accounting for the reduced functions of the aging brain [125]. Neurogenesis in adult primates, a recurring and crucial topic of primate neuroscience, has been comprehensively investigated through sc/snRNA-seq transcriptomic data accompanied by sufficient immunostaining evidence [126, 127]. Larger-scale transcriptomic studies have also focused on the diversity of glial cells, including oligodendrocytes and astrocytes [128], which exhibit developmental and metabolic regulation by neuronal activity in the developing human cerebral cortex [129, 130]. This result indicates that the balance of the interaction between glial cells and neurons is important for the normal development of the primate brain.

### Transcriptome Changes of Neuropathological State

Single-cell and single-nucleus transcriptomic analyses enable the exploration of cell type composition, transcriptomic modifications, and their role in neurological diseases. These modifications, particularly in critical genes and pathways, regulate disease progression and offer therapeutic opportunities [131, 132]. Such analyses have been extensively used to study various primate neurological disorders, establishing cellular taxonomies, identifying vulnerable cell subpopulations with risk genes, and shedding light on pathogenesis mechanisms and potential therapeutics [133–139]. In this review, we dived into the detailed research on Alzheimer's disease (AD), autism spectrum disorder (ASD), and multiple sclerosis (MS).

AD is a progressive neurodegenerative disorder characterized by memory loss, cognitive decline, and executive dysfunction [140–142]. Analyses of single nuclei from the prefrontal cortex of individuals with AD have identified distinct neuronal and non-neuronal types with pathological gene expression associated with myelination, inflammation, and neuron survival. Disease-associated changes are highly cell-type specific, with some genes (*HSP90AA1* and *HSPA1A* involved in protein folding) universally upregulated in late stages [143]. Transcriptional and pathological differences between sexes have also been reported [139]. Understanding cell-type-specific gene networks and transitions is crucial for unraveling AD pathogenesis. Integrated analysis of transcription factors and AD risk loci have revealed drivers of cell-type-specific transitions. It highlights the repression of AD risk genes in oligodendrocyte progenitor cells, astrocytes, and their upregulation in microglia [144–146]. Cell-type-specific vulnerability is a fundamental feature of neurodegenerative diseases in which different cellular populations show a gradient of susceptibility to degeneration. Abundant molecular signatures identified by snRNA-seq provide an unprecedented chance to characterize the specifically vulnerable neuronal subpopulations at the molecular level. In transcriptomic analysis of AD, *RORB* (RAR Related Orphan Receptor B) has been identified as a marker of selectively vulnerable excitatory neurons. At the same time, the downregulation of genes involved in homeostatic functions is used to characterize vulnerable astrocyte subpopulations [147, 148]. Recent multimodal methodology has identified potential AD treatments [149].

ASD is a neurodevelopmental condition impacting interaction and communication [150]. Recent research investigating 11 cortical areas in individuals with ASD and neurotypical controls has revealed widespread transcriptomic changes across the cortex in individuals with ASD. The findings exhibit an anterior-to-posterior gradient, with the most significant differences in the primary visual cortex. These differences coincide with reduced typical transcriptomic differences between cortical areas in neurotypical individuals [151]. In relation to the cell-type-specific molecular changes associated with ASD, Velmeshev et al. found that synaptic signaling in upper-layer excitatory neurons and the molecular state of microglia are preferentially affected in ASD. Moreover, the dysregulation of specific groups of genes in cortico-cortical projection neurons has been found to correlate with the clinical severity of ASD, as expected [48].

Oligodendrocytes are implicated in the pathogenesis of MS, a multifocal inflammatory disease affecting cortical areas [152]. SnRNA-seq has demonstrated different functional states of oligodendrocyte subpopulations in MS tissue and has identified selectively vulnerable neuronal subpopulations, stressed oligodendrocytes, reactive astrocytes, and activated microglia associated with the progression of MS lesions [153, 154]. Overlapping transcriptional profiles between MS and other neurodegenerative diseases suggest shared mechanisms and potential therapeutic approaches [155].

## Linking Transcriptomes with Morphological and Electrophysiological Phenotypes

Single-cell or single-nucleus sequencing data provide valuable insights into the transcriptomic types (t-types) of homology across species [15, 90]. However, it cannot obtain the morphological and electrophysiological properties of the morpho-electrical-transcriptomic types [56, 59]. Patch-seq is a revolutionary technology that can simultaneously acquire the morphology, electrophysiology, and transcriptome of single cells, which are valuable resources to establish a multimodal atlas. Patch-seq is a modification of regular patch-clamp recording. It has been applied to record from cultured cells and acute brain sections *in vitro* [56–58, 60], and the recorded cells are labeled with dye for subsequent morphological reconstruction. After electrophysiological recording, most of the cytoplasmic contents are aspirated (generally including the nucleus) and transferred to an individual tube containing a lysis buffer followed by a standard single-cell or single-nucleus RNA-seq protocol (Fig. 1D−G). This powerful multimodal approach provides valuable resources for advancing our understanding of primate neuroscience.

Patch-seq techniques have been successfully implemented in the primate cortex [16, 59, 61, 62, 64, 90]. Patch-seq-sampled data from neurons across human cortical layers 1, 2, 3, and 5 have been mapped to a human transcriptomic cellular reference atlas and assigned electrophysiological and morphological features to the mapped t-types [16, 61, 64]. This multimodal analysis has identified human-specific double bouquet cells, mapping to two cortical GABAergic somatostatin (SST) t-types (*SST CALB1* and *SST ADGRG6*) [62]. Studies on the human cortex have revealed higher divergence in the upper-layer neocortex compared to mice [16], whereas the cell density in layers 2/3 is lower in humans than in mice [16, 61, 156]. Among human L1 interneurons, subclasses defined by their transcriptomes exhibit similarly distinct morpho-electrical phenotypes. Two human cell types with specialized phenotypes have been identified (*MC4R* rosehip cells and the bursting *PAX6 TNFAIP8L3* t-type) [61]. Indeed, observing a 'rosehip' cell type in human and not mouse neocortex emphasizes the importance of studying human L1 to uncover potential species-specific specializations [12].

In addition, the supragranular layer of the human neocortex exhibits increased diversity in glutamatergic neuron types, predominantly found in layers 2 and 3. Five human supragranular neuron t-types (*LTK*, *GLP2R*, *FREM3*, *CARM1P1*, and *COL22A1*) have corresponding morphology, physiology, and transcriptome phenotypes. The more superficially located *LTK*, *GLP2R*, and *FREM3* types are homologous to the mouse supragranular IT types. The more deeply located *CARM1P1* and *COL22A1* types do not have direct counterparts in the mouse supragranular neocortex. Instead, they exhibit the closest transcriptomic similarity to infragranular mouse IT types [16]. These results suggest an increased diversity of deep L3 neurons in humans. The deep portion of layer 3 contains highly distinctive cell types, two pyramidal cell types (*FREM3+* and *CARM1P1+* transcriptomic cell types [15]) expressing neurofilament protein SMI-32 (encoded by the *NEFH* gene), which labels long-range projection neurons in primates that are selectively depleted in AD [157, 158], providing a promising entry to study the pathological mechanism and explore potential therapeutic options.

In contrast to the well-documented diversity of supragranular-layer excitatory neurons across regions and species, studies addressing the variability of deep-layer excitatory neurons, such as ET, IT, and CT neurons, are limited [90]. Axonal projections to lower brain regions predominantly originate from layer 5 (L5) ET neurons. L5 ET neurons exhibit unique morpho-electric properties, gene expression patterns, local synaptic connections, long-range afferents, and neuromodulatory responses, as primarily described in rodents. L5 ET neurons are traditionally characterized by thick apical dendritic tufts in layer 1, while L5 IT neurons have thinner or tuftless dendrites. In addition, L5 ET neurons strongly express hyperpolarization-activated cyclic nucleotide-gated (HCN) channels, likely contributing to their strong dendritic electrogenesis [64]. Still, in rodents, the HCN conductance tends to dampen dendritic electrogenesis. The differences in HCN expression between L5 ET and IT neurons may not be the primary reason for the differences in electrogenesis [159]. Further interrogations are necessary. These different properties of L5 ET and IT neurons contribute to distinct aspects of perception and behavior. Patch-seq analysis of L5 neurons in the primary motor cortex of both mice and macaques has revealed that macaque and human Betz cells are homologous to the thick-tufted L5 ET neurons in mice but exhibit species-specific differences in morphology, physiology, and gene expression. Macaque and human Betz ET neurons have specialized suprathreshold properties, such as biphasic firing patterns evoked by prolonged suprathreshold current injection [26]. Macaque and human L5 ET neurons are notably larger and possess long "taproot" basal dendrites, characteristic of the iconic Betz cells [160].

Gene expression patterns shape the electrophysiological and morphological phenotypes. Patch-seq provides a multimodal analysis strategy to identify potential molecular markers or pathways that predict the phenotype of single neurons [161, 162]. Using Patch-seq combined with Weighted Gene Co-expression Network Analyses of single human neurons in culture, certain gene clusters are correlated with neuronal maturation as determined by electrophysiological characteristics, and a list of candidate genes has been identified that have the potential to serve as biomarkers of neuronal maturation [163]. For example, Patch-seq recording from human induced pluripotent stem cell (iPSC)-derived astrocytes and neurons has revealed a continuum of low- to high-function electrophysiological states. Furthermore, a novel biomarker, *GDAP1L1,* effectively identifies the high-functioning neurons. These biomarkers facilitate the classification of neurons based on their functionality and enable the stratification of functional heterogeneity [161].

Subtle differences in the transcriptome may profoundly affect neuronal morphology and function [164]. Patch-seq links transcriptomes with phenotypes such as morphology and electrophysiology. This allows for targeted studies on specific neuronal populations based on factors like anatomical location, functional properties, and lineages. Patch-seq also facilitates studies on the molecular basis of morphological and functional diversity [165]. We can gain new insights into neurons by using Patch-seq to correlate transcriptomes with morphological characteristics, neuronal locations, and projection patterns. The acquisition and integration of transcriptome information is essential to neuronal classification.

## Dissecting Local Connectivity Principles in Primate Cortical Areas

Understanding the functional mechanisms of primate cortical areas requires a comprehensive investigation of their cellular and synaptic organization. To enable a detailed understanding of microcircuits, multicell whole-cell patch-clamp recordings still represent the gold standard method [166, 167]. This method reliably detects unitary excitatory and inhibitory synaptic connectivity with its submillisecond and subthreshold resolution [168]. Increasing the number of simultaneously recorded neurons can considerably increase the number of probed synaptic connections and generate larger sample sizes from fewer experiments. For example, if the number of simultaneously patched neurons reaches eight in single slices, 56 potential connections will be tested. It is important to achieve relatively high throughput due to the limited availability of primate samples. By using multicell whole-cell patch-clamp recordings, simultaneous recording of multiple neurons becomes possible. This approach significantly enhances the number of monosynaptic connections tested per experiment, enabling us to explore the principles of connection between different cells (Fig. 1H, I) [83, 169–173].

The recent application of multicell patch-clamp technology to the study of the primate brain has considerably expanded our knowledge of its microcircuits. Recurrent excitatory connectivity is thought to be important in behavior [174] and disease [175], and has also been identified as a common feature in computational models of cortical working memory, receptive field shaping, attractor dynamics, and sequence storage [176–180]. Although there is a wide range of reported rates of recurrent connectivity among excitatory neurons in rodents [173, 181, 182], evidence has shown that the human cortex possesses a higher recurrent excitatory connectivity rate and mean amplitude, which might contribute to the related functional difference across species [183, 184]. The higher mean amplitude and other excitatory postsynaptic differences indicate stronger synaptic connectivity within the human cortex, which is explained by the larger presynaptic active zones and postsynaptic densities that may allow a higher release probability as well as more neurotransmitter release and binding [185, 186].

A more comprehensive survey of intralaminar connectivity has been applied to investigate all cortical layers detailing the connectivity atlas, and the analysis includes synaptic dynamics between layer-defined pyramidal neurons and inhibitory neurons, greatly increasing our understanding of primate neural microcircuits [187]. In this study of the connectivity between layer-defined neuronal types, the connectivity probability among layer 4 was nearly absent in the human cortex. At the same time, it was high in the mouse cortex. Moreover, disynaptic inhibition in the human cortex, which was not detected in the mouse cortex, was found between confirmed spiny pyramidal cells that were unidirectional, originating in layer 2 and targeting other layer 2 or layer 3 pyramidal cells. Consistent with previous results, the connectivity rates were estimated to decline with increasing distance but at a slower speed than in rodents. The unique characteristics of the human circuit findings regarding alterations in synaptic dynamics may help explain the complexity of information in the human cortex.

The morphologies of neurons are diverse. Their dendritic and axonal projections provide additional information for investigating the local connectivity. Recent advances in patch-clamp-based techniques and solution formulations have significantly improved the quality of neuron reconstruction [63], establishing a framework for the morphological classification of rodent neurons [60, 173]. In addition, high-throughput information can be obtained through the advances in imaging and cell labeling techniques, such as superresolution hopping probe ion conductance microscopy, a variant of scanning ion conductance microscopy [188–190] and viral tracers or transgenic animals (e.g., Cre-driven lines or tetracycline-controlled transcription factors for labeling specific neurons) [57, 191–195]. The morphological classification of inhibitory and excitatory neurons in rodents can serve as a preliminary reference for establishing the morphological classification of brain cells in primates. This reference can expedite the generation of a comprehensive connectivity atlas. By leveraging the knowledge and techniques developed in rodent studies, researchers can accelerate the mapping of neural circuits in primates, providing valuable insights into brain connectivity and function.

## Leveraging the Core Advantages of the Above Technologies to Generate a Multimodal Atlas

### The Advantages of these Three Technologies

**Fig. 2:**
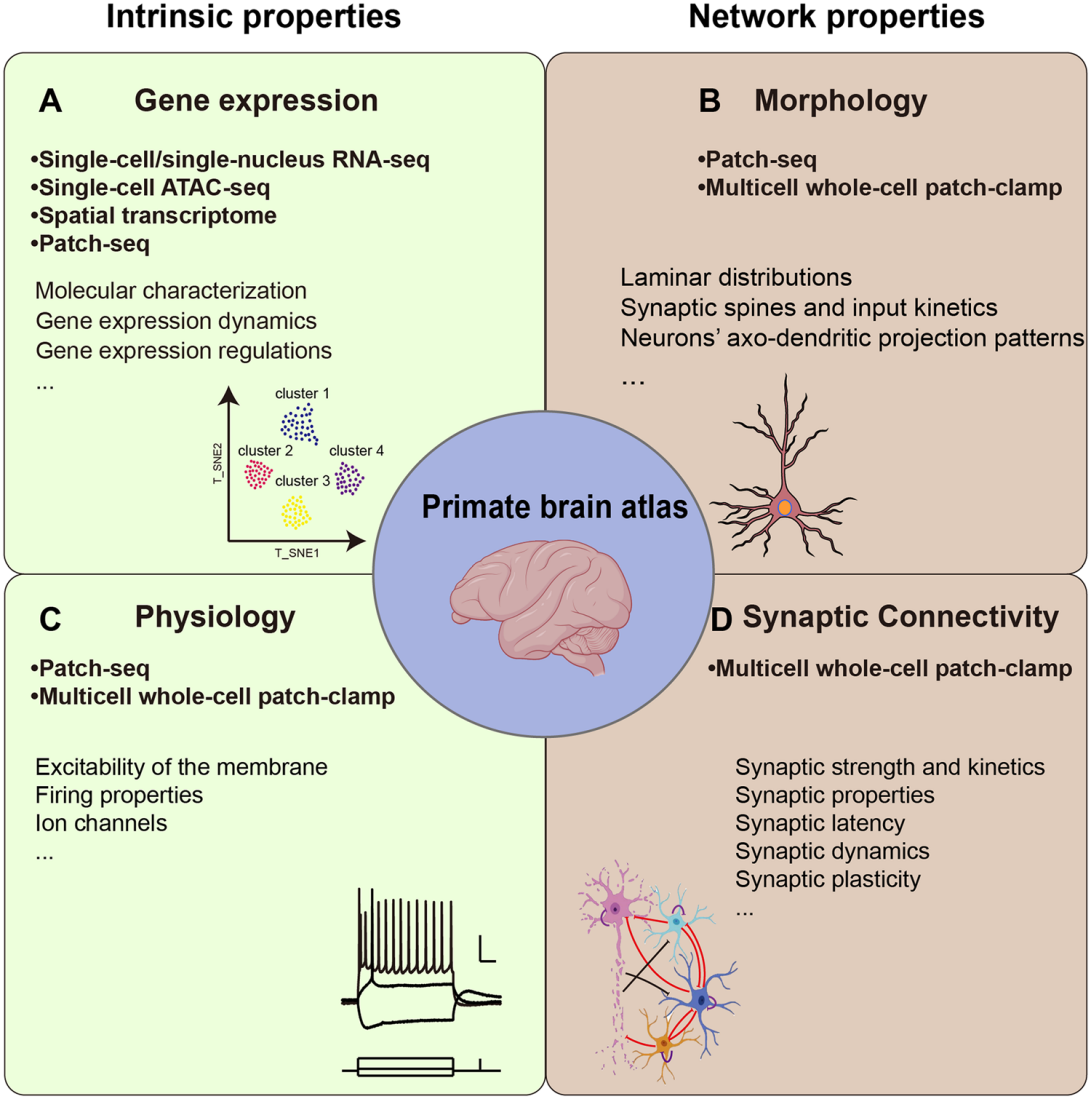
Framework for depicting the primate brain atlas. Four modalities are illustrated. **A** Gene expression. Using single-cell/single-nucleus RNA-seq, single-cell ATAC-seq, spatial transcriptome, and Patch-seq, we can obtain the molecular characterization, gene expression dynamics, and gene expression regulation of each neuron. **B** Morphology. Patch-seq and multicell whole-cell patch clamps can provide laminar distributions, synaptic spines, input kinetics, and axo-dendritic projection patterns. **C** Physiology. Using Patch-seq and multicell whole-cell patch-clamp can provide the phenotypes of the neurons, such as the excitability of the membrane, firing properties, and ion channels. **D** Synaptic connectivity. Multicell whole-cell patch-clamp measures synaptic strength and kinetics, synaptic properties, synaptic latency, synaptic dynamics, and synaptic plasticity. Drawings of primate brains were created with BioRender.com.

The primate brain is organized into cortical areas responsible for different functions. Different types of cells within cortical areas with specific transcriptomic, morphologic, and electrophysiologic profiles establish synaptic connectivity following principles. During development, aging, and disease, the electrophysiology, morphology, and connectivity phenotypes of cell types are simultaneously affected and undergo different degrees of adaptation under the control of transcriptomic modifications. As in the mouse, the single-cell data on the morphology, electrophysiological properties, and gene expression patterns in the primate brain need to be integrated [196] for comprehensive knowledge of functional mechanisms in primate cortical areas. Single-cell and single-nucleus RNA-seq provide robust cell-type classification data to generate a transcriptomic reference atlas that can be used to illustrate the cellular composition of cortical areas and to perform bioinformatics analysis to explain the modifications that occur during physiological and pathological states (Fig. 2 A). Patch-seq results, which can be mapped to the transcriptomes provided in the above transcriptomic reference atlas, establish morphological and electrophysiological annotations for each cell type. Based on the advances in multicell patch-clamp recordings with detailed morphological reconstruction, especially the morphology of inhibitory neurons [60, 173], local connectivity principles among morphology-based neuronal types will be delineated in primates as in rodents (Fig. 2B, C). The strong correspondence between morphological and electrophysiological phenotypes of cells [16, 64] might be a theoretical basis for mapping the local connectivity principles to the above transcriptomic atlas with morphological and electrophysiological annotations. To this end, a primate brain atlas that integrates the datasets from the above three technologies can describe the transcriptomic, morphological, and electrophysiological profiles of each type and the local connectivity principles. This atlas will play a crucial role in investigating the cortical regions in primates and significantly contribute to research on degenerative diseases in primates.

### Atlases with Multiple Dimensions Resolve Novel Neuroscience Questions

Extensive data on the applications of the three technologies are now being obtained from primate brains to generate a primate brain atlas with multiple dimensions within cortical areas. The atlas will describe the multiple profiles, including transcriptomes, electrophysiology, and morphology, as well as the local connectivity principles, in each cell type within primate cortical areas (Fig. 2). The organizational differences within homologous cortical areas could explain the higher complexity of functions in primates than in rodents. When establishing the above atlas, larger-scale transcriptome analysis might be used to identify species-specific cell types with distinct gene expression patterns [16, 64] and neuronal biology [90], and the local connectivity patterns of a selected cell type could then be comprehensively studied by the applications of Patch-seq and multicell patch-clamp (Fig. 2D).

Although studies show a surprising conservation of basic transcriptomic cell types across cortical areas between primates and rodents [10, 15, 99, 100], modifications of the primate neuronal profiles of transcriptomes, electrophysiology, morphology, and connectivity patterns might contribute to the more complex brain functions. In rodent studies, the integration of data from Patch-seq and sn/scRNA-seq has allowed for a comprehensive multimodal analysis. This approach successfully identified distinct morphology-electrophysiology-transcriptome types, showcasing unique neuronal properties. Moreover, these types can form continuous and correlated transcriptomic and morphological electrical landscapes within their respective families [72, 197]. This mutual predictability not only helps to predict the functional, morphological, or transcriptomic state based on one or two distinct neuronal properties but also provides promising potential for integration with experimental data from other technologies. In pathological studies, since large-scale transcriptome analysis has identified vulnerable cell types [139, 151, 153, 154], Patch-seq can be used to test the potential loss or gain changes in the transcriptomic, electrophysiological, and morphological aspects of these vulnerable cell types, and multicell patch-clamp can be used to detect the loss or gain changes of local connectivity involving the selected cell types. Gene expression pattern analysis not only explains the molecular mechanisms of functional changes but also supplies lists of marker genes of cell types to develop genetic manipulation tools, which might serve in the applications of Patch-seq and multicell patch-clamp in primates. Moreover, Patch-seq and multicell patch-clamp can be used to verify the therapeutic effectiveness of targeting drug candidates provided by the transcriptome analysis [48, 139, 198, 199].

## Challenges and Future Efforts

These technologies have considerable potential to address crucial questions in primate neuroscience. Each of the above three technologies has already made important contributions to advances in primate neuroscience. Establishing a primate brain atlas with multiple dimensions by combining the advantages of the three technologies is extraordinarily promising. Still, several important challenges remain.

Due to the scarcity of primate tissues, especially those from primate models of neurological diseases, establishing a detailed primate brain atlas is challenging. In the future, integrating Patch-seq and multicell patch-clamp techniques with non-invasive imaging methods, such as magnetic resonance imaging and diffusion tensor imaging, offers a promising avenue for obtaining detailed structural and connectivity information about the primate brain *in vivo* [200].

Secondly, implementing Patch-seq and multicell patch-clamp techniques faces challenges due to their relatively low throughput. Future efforts should focus on developing automated and high-throughput methods for Patch-seq and multicell patch-clamp techniques. By streamlining the experimental workflow and optimizing protocols, more cells can be processed within a shorter time frame, allowing for a more comprehensive analysis of the diverse cell types in the primate brain.

Moreover, commonly used genetic manipulation tools are mainly used in rodents and organotypic section cultures in humans and non-human primates [201–205]. Future development of genetic manipulation tools, such as expressing fluorescent proteins to label specific neurons or using optogenetic approaches *in vivo* to dissect circuit connections, could reveal patterns of synaptic connectivity in different species, including non-human primates [193, 206–210].

Finally, to obtain transcriptome information from multicell patch experiments, future research should prioritize the development of techniques that enable the simultaneous patching of multiple cells, extraction of nuclei for RNA retrieval, and concurrent acquisition of morphological and electrophysiological information. For example, spatial single-cell analysis of multiple genes using multiplexed error-robust fluorescence *in situ* hybridization (MERFISH) has generated a molecularly-defined and spatially-resolved cell atlas [211]. Integrating Patch-seq with multicell patch-clamp and single-cell sequencing technologies is immensely important in establishing comparative connectivity maps across different states or cortical regions. By combining these techniques, researchers can establish comparative connectivity maps, enabling the study of neural circuitry across different conditions or regions of the cortex. This integration paves the way for a deeper understanding of brain function and connectivity.

In conclusion, a rodent transcriptomic cell atlas has become available with morphological and electrophysiological annotation [58, 72, 197, 212] and delineating the local connectivity principles [173, 213]. A primate neocortex transcriptomic atlas has been established, while the morphological, electrophysiological, and connective properties are mostly absent. Detailed interrogation of these neurons indicates a higher diversification of excitatory neurons in the supragranular layer. Further studies on a broader range of brain areas and cell types, together with a cellular atlas describing developmental, aging, and disease states, are necessary to fully understand the molecular and neuronal basis of the augmented cognitive and behavioral capabilities of higher primates.
